# Using Program Data to Improve Access to Family Planning and Enhance the Method Mix in Conflict-Affected Areas of the Democratic Republic of the Congo

**DOI:** 10.9745/GHSP-D-17-00365

**Published:** 2018-03-21

**Authors:** Lara S Ho, Erin Wheeler

**Affiliations:** a International Rescue Committee, Washington, DC, USA.; b International Rescue Committee, New York, NY, USA.

## Abstract

Unmet need for family planning in the conflict-affected area of eastern Democratic Republic of the Congo (DRC) has been reported to be as high as 38%, and women in such conflict settings are often the most at risk for maternal mortality. The International Rescue Committee implements the Family Planning and Post-Abortion Care in Emergencies program in 3 provinces of eastern DRC to provide women and couples access to family planning, including long-acting reversible contraceptives (LARCs). This article presents routine program data from June 2011 through December 2013 from 2 health zones as well as results from a qualitative assessment of family planning clients and of male and female non-users, conducted in 2013. It then describes how these findings were used to make program adjustments to improve access to family planning services and client informed choice and assesses the effects of the program design changes on family planning uptake and method mix using routine program data from January 2014 through December 2016. Between 2011 and 2013, 8,985 clients adopted family planning, with an average 14 clients adopting a method per facility, per month. The method mix remained stable during this period, with implants dominating at 48%. Barriers to uptake identified from the qualitative research were both supply- and demand-related, including misconceptions about certain modern contraceptive methods on the part of providers, users, and other community members. The program implemented several program changes based on the assessment findings, including clinical coaching and supportive supervision to improve provider skills and attitudes, introduction of immediate postpartum insertion of the intrauterine device (IUD) and the levonorgestrel-releasing intrauterine system (LNG-IUS), and behavior change communication campaigns to raise awareness about family planning. After these program changes, the mean number of clients adopting modern family planning per facility, per month increased from 14 to 37 and the percentage of family planning adopters choosing LARCs increased from 50% to 66%. While implants continued to be the most dominant method, reaching 60% of the method mix in 2016, the percentage of clients adopting IUDs increased each year, from 3% in 2014 to 13% in 2016. In total, 39,399 clients started family planning methods during the post-program design change period (2014–2016). Our experience in eastern DRC demonstrates that women and their partners affected by conflict want family planning, and that it is feasible to deliver the full range of modern contraceptive methods when programs are adapted and sensitive to the local context.

## Abstract (precis)

Analysis of program data and a formative assessment informed several program changes, including improved coaching and supportive supervision, introduction of postpartum IUDs and the levonorgestrel-releasing intrauterine system, and enhanced behavior change communication. These changes substantially increased family planning adoption, from a monthly average of 14 adopters per facility to 37 per facility. Implants continued to be the most popular method, but the percentage of adopters choosing the IUD increased from 2% in 2012 to 13% in 2016, and it was the most popular method among postabortion care clients.

## BACKGROUND

All too often, the humanitarian sector marginalizes family planning as impractical or unimportant in humanitarian crises. An analysis of humanitarian funding appeals over a 10-year period found that less than 15% of health and protection proposals included family planning—the second lowest percentage among all reproductive health services.^1^ This lack of investment is evident in family planning service availability. A study of the availability of reproductive health services in 3 crisis-affected settings in sub-Saharan Africa found that only 16% of 63 health facilities assessed were capable of providing comprehensive family planning services.^2^

Unintended pregnancies can have a negative impact on the lives of women and girls living in under-resourced, conflict-affected countries such as the Democratic Republic of the Congo (DRC), where maternal mortality increased from an estimated 543 deaths per 100,000 live births to 846 deaths per 100,000 live births between 2007 and 2013.^3^ Unmet need for family planning in conflict-affected areas such as eastern DRC is as high as 38%.^3^ While fertility desires among crisis-affected populations may be affected in the short-term by acute conflict, the determinants of fertility in these settings tend to be similar to those in stable, low-resource settings.^4^

Unmet need for family planning in conflict-affected areas such as eastern DRC is as high as 38%.

The protracted conflict in eastern DRC began during the 1994 Rwandan genocide and has continued beyond the First and Second Congo Wars from 1996 to 2003. Decades of violence involving dozens of armed groups have led to periodic large-scale population displacement. In 2012, the rebel group M23 destabilized both North and South Kivu until their defeat in 2013.^5^^,^^6^ More recently, political turmoil caused by the delayed 2016 presidential elections and accompanying economic crisis have exacerbated tensions and conflict in eastern DRC.^7^ In 2017, 4.1 million people were displaced across the country.^8^ Civilians have frequently been targeted by rebel and government armed groups, whose human rights abuses are well-documented.^9^

The complex emergency combined with poor governance has led to a weak health system, a vulnerable population, and high fertility and poor health outcomes among women. The total fertility rate in the country is 6.6 children per woman. In South Kivu, 22% of women have an unmet need for family planning, only 7.9% of women use a modern method of contraception, and less than half of those using modern methods use long-acting or permanent methods, while in Bas Congo 17.2% of women use a modern method.^3^ Across the DRC, long-acting reversible contraceptives (LARCs) usually make up less than half of modern methods used.

**FIGURE 1.**
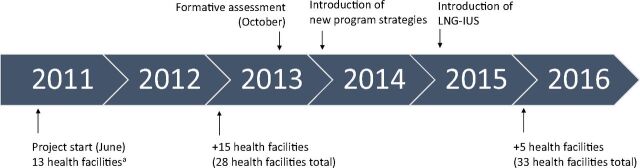
Project Timeline, Health Zones A and B in South Kivu, Democratic Republic of the Congo Abbreviation: LNG-IUS, levonorgestrel-releasing intrauterine system. ^a^ Includes 2 facilities from a different health zone in South Kivu than the other health facilities reported in this article.

Since 2011, the International Rescue Committee (IRC) has been implementing a project in the DRC to increase access to voluntary contraception, in particular LARCs, and postabortion care (PAC). At the start of the project in June 2011, IRC supported 42 Ministry of Public Health (MoPH) facilities in North Kivu, South Kivu, Kasai Occidentale, and Province Orientale. As of December 2017, the project supported 61 MoPH facilities in 6 health zones in North Kivu, South Kivu, and Tanganyika provinces. In South Kivu, the project supported 13 MoPH facilities in 2 health zones from June 2011 to December 2012, 28 MoPH facilities from January 2013 to December 2015, and 33 MoPH facilities from January 2016 to present (Figure 1). The project supports the MoPH to provide a range of contraceptive methods, including long-acting, short-acting, and permanent methods, to people living in areas affected by conflict. Support includes competency-based training, supportive supervision, procurement and supply chain management, data management and use, and community mobilization. Family planning commodities are subsidized so that they are free to users and consistently available.

Family planning experts have not defined an ideal method mix but agree that it can be a useful indicator of program quality. If one method makes up a disproportionally high or low percentage of the method mix, this may be an indication of poor quality of counseling or provider preference for a particular method, thereby excluding some methods from equal consideration by clients and limiting practical availability of all methods. Evidence demonstrates that provider attitudes and skills influence which contraceptive methods are offered and to whom.^10^^–^^14^ Sullivan and colleagues defined a skewed method mix among current users of contraception as one in which a single method is used by 50% or more of women using contraception.^15^ While this definition was developed for population-level data, it is a useful standard that contraception programs can apply to service statistics to determine when further attention to program quality and client informed choice may be needed.

From June 2011 to May 2013 in the 2 health zones in South Kivu supported by IRC, 48% of clients who adopted family planning methods chose an implant while just 2% of clients chose an intrauterine device (IUD). This same trend toward implants was also noted by Curry and colleagues, who analyzed a similar program in the DRC.^16^ This method skew among adopters led us to conduct an investigation in 2013 to better understand the program factors influencing both family planning uptake and method mix so that we could identify and implement targeted activities to improve program quality. In particular, we wondered why, given the clear preference for long-acting methods, so few clients accepted IUDs in our program while other programs in eastern DRC demonstrated higher IUD uptake.^16^ This investigation included more robust analysis of existing facility-level data and formative research on knowledge, perceptions, and attitudes toward family planning among users and non-users. The purpose of this article is to describe: (1) the results of this investigation, (2) the changes in program design implemented based on these results, and (3) the effects of this improved program design on project indicators in the 2 health zones in South Kivu.

From 2011 to 2013, 48% of clients in South Kivu who adopted family planning methods chose implants while just 2% chose IUDs.

## METHODS

### Analysis of Program Monitoring Data

The project collected data on family planning and PAC clients, including the number of women and men adopting family planning (defined as those who start a method of contraception for the first time, who start a method after 6 months of non-use, or who switch to a new method of contraception) disaggregated by method, month, and facility. The methods reported are IUDs, implants, oral contraceptive pills, injectables, tubal ligations, and vasectomies. The number of clients adopting traditional methods and condoms is not collected by the project. An important component of PAC is counseling on and receipt of family planning. The definition used for PAC clients who adopted family planning varied between 2011 and 2016. From June 2011 to March 2015, those PAC clients who adopted a contraceptive method up to 2 weeks after PAC were included in the number of PAC clients who adopted family planning. From April 2015 to December 2016, however, only those PAC clients who adopted family planning before discharge were counted.

The data collected and analyzed were from MoPH registers and monthly reporting forms. Health facility staff completed a monthly reporting form, which was submitted to the Health Zone Management Team and to IRC. Until 2015, IRC staff at the provincial level would then enter these data into Microsoft Excel spreadsheets, which were then aggregated into a country-level Excel database for analysis at the national and global level. In 2015, the organization introduced a DHIS 2 platform for staff to enter data directly into the global project database for analysis at the country and headquarters levels.

Over the project period, project staff coached MoPH providers to ensure accurate recording of service delivery data and together conducted regular reviews of both data quality and trends. From 2012 to 2015, a third-party evaluator conducted annual program reviews, which further validated the accuracy of the data and fostered an environment of data use. The data presented here are from 2011 to 2016 from the 2 health zones where qualitative data collection took place.

### Formative Research to Design Program Strategies

We conducted formative research to inform program design in October 2013. Based on analysis of program data, 6 health facilities from the 2 health zones were purposively selected to represent relatively higher and lower uptake of family planning in general and IUDs in particular (Table 1). At least 2 providers at each of the selected facilities had received competency-based training on family planning service provision, including IUD insertion and removal, and had placed at least 2 IUDs in the 6 months preceding data collection, suggesting that the providers had minimum capacity to do so.

**TABLE 1.** Sampling of Health Facilities for Formative Research

Health Zone	Facility	Reason for selection
A	1	Relatively high proportion of clients accepting IUDs
	2	Relatively low proportion of clients accepting IUDs
	3	High number of clients accepting contraceptive methods, relatively low proportion of clients accepting IUDs
B	4	High number of clients accepting contraceptive methods, relatively high proportion of clients accepting IUDs
	5	High number of clients accepting contraceptive methods, relatively low proportion of clients accepting IUDs
	6	High number of clients accepting contraceptive methods, relatively low proportion of clients accepting IUDs

Abbreviation: IUD, intrauterine device.

Data collection included 23 semi-structured interviews with clients who had adopted short-acting or long-acting methods (both implants and IUDs) at one of the purposively selected health facilities. In total, 24 women were purposively selected for interviews, 4 at each facility, but 1 did not appear at the planned time of the interview. We also conducted 5 focus group discussions with 40 male non-users of contraception and 5 focus group discussions with 38 female non-users of contraception, which included free-listing exercises.

Inclusion criteria for users by facility consisted of:

- Two women who had adopted an IUD within the previous 6 months
- One woman who had adopted an implant within the previous 6 months
- One woman who had adopted an injectable within the previous 6 months

Inclusion criteria for non-users consisted of:

- Women who were non-users of family planning in the previous 5 years and had at least 5 children
- Partners of women meeting the above criterion

Users were purposively selected from registers, whereas non-users were recruited through community-based organizations in the facility catchment areas.

Data were collected from October 2–10, 2013. The lead author and 1 local staff member trained 4 data collectors with previous qualitative research experience and supervised the data collection; the training lasted 2.5 days. Interviewers obtained oral informed consent prior to each interview. IRC also received approval for the activities from the Health Zone medical directors. Interviews were conducted in Kiswahili or Mashi, transcribed by the interviewers, and translated into French.

The trainers conducted preliminary analysis of field notes immediately after data collection through multiple readings and discussion with data collectors to identify immediate remedial actions. The lead author conducted additional analysis in Atlas.ti using open coding to generate themes. At the same time, the project staff member used Microsoft Word to categorize passages according to emerging themes. The lead author and the project staff member then shared their findings to harmonize analyses. During the focus group discussions, reasons for using or not using family planning were listed and ranked by group consensus. In order to understand which reasons were more important than others, salience scores were calculated by averaging the percentile ranks for each reason across the groups. The percentile rank for items listed by each group were calculated using the formula (total number of items in list-rank order of A)/(total number of items in list).^17^

## RESULTS FROM THE 2013 INVESTIGATION

### Quantitative Data on Contraceptive Users

**FIGURE 2.**
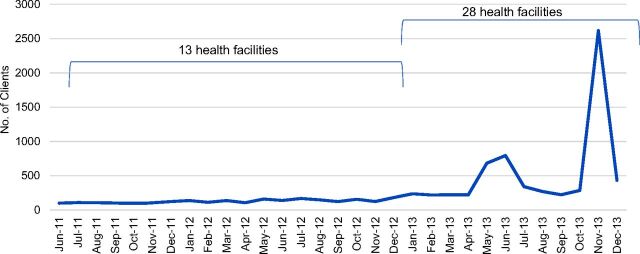
Number of Clients Adopting Family Planning Methods, Health Zones A and B in South Kivu, Democratic Republic of the Congo, June 2011 to December 2013

From June 2011 through December 2013, the average number of clients who adopted family planning methods per facility, per month was 14 in the 2 health zones, with 8,985 clients in total adopting family planning methods during the 30-month period (Figure 2). Among 1,100 clients who received PAC, 29% adopted a family planning method. The organization conducted 2 large social and behavior change communications (SBCC) campaigns toward the end of the period, during which time spikes in clients starting family planning methods were observed.

On average, 14 clients adopted family planning methods per facility, per month between 2011 and 2013.

**FIGURE 3.**
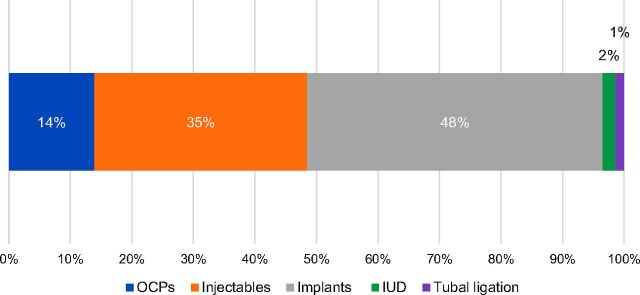
Contraceptive Method Mix Among Clients Adopting Family Planning Methods, Health Zones A and B in South Kivu, Democratic Republic of the Congo, June 2011 to December 2013 Abbreviations: OCP, oral contraceptive pill; IUD, intrauterine device.

The contraceptive method mix among all clients adopting modern family planning methods as well as PAC clients who adopted family planning remained stable during the period, with contraceptive implants and injectables most frequently chosen in both zones, followed by pills, IUDs, and tubal ligation (Figure 3).

### Qualitative Data From Contraceptive Users

The 23 respondents who had adopted family planning methods ranged in age from 21 to 53 and had 3 to 11 children.

#### Factors and People That Influence Family Planning Decisions

The primary reasons for choosing to adopt family planning were respondents' own health and the economic burden of raising multiple children. Several respondents spoke about being able to space pregnancies, provide better care for their existing children, and be healthy so that they could continue working. One respondent explained:

*I saw that in giving birth each time, we never accomplished a project because even if we save a bit of money today and the mother gets pregnant, we're obliged to prioritize the pregnancy and let everything else fall to the wayside.*

Husbands were the most influential in deciding whether a couple adopted family planning, followed by health care providers.

Husbands were the most influential in deciding whether a couple adopted family planning, followed by health care providers. One woman reported:

*It was only at the 8th birth that I conceived 6 months after giving birth [to my 7th child] … and then I was poisoned. My body with many weaknesses required rest. That's when my husband adopted the idea of accessing family planning.*

Many women discussed family planning with their friends and supported other women in deciding to use family planning after adopting a method themselves. Although a number of women reported having side effects from family planning, these were not significant enough to prevent them from continuing to use family planning.

#### Sources of Information

Women most often cited health centers as their primary source of information on family planning, including during antenatal and well child visits. According to one woman:

*I learned about family planning during antenatal care at the health center. The nurse spoke about the benefits of family planning. I was interested during my 12th pregnancy; in the 9^th^ pregnancy I had a still birth and almost died.*

Next, women followed the advice of their friends or other women in the community. Other sources of information mentioned were sensitization sessions in community-based organizations or by community health workers, or information shared by the church. Often women received information from multiple sources before choosing to adopt family planning.

#### Quality of Services

As described earlier, health centers are the principal entry point for family planning services. Descriptions of care received suggested variation in the quality of services provided. Even if the majority of users were informed about all the methods available, some of the information the clients reported receiving revealed either gaps in or misunderstanding about the counseling provided. For instance, one respondent said that she had heard of an IUD but had never seen one, despite the fact that all providers had been provided with displays of all methods to be used during counseling sessions. Another reported that the nurse had told her to return to the health center to insert a new IUD after 5 years of using her current IUD, when in fact at the time all IUDs provided by the project were copper-T IUDs, which are effective for at least 10 years.

Some of the information the clients reported receiving from providers revealed either gaps in or misunderstandings about the counseling provided.

In addition, it seemed that in some cases providers were selecting methods for clients, while they had been trained to provide counseling that supported informed choice by the client. Women reported being told that young women who had not yet had a child could not use an IUD. Another was counseled to use an injectable method rather than a LARC or permanent method even though she wanted no more children. One provider refused a respondent's request to switch from a short-acting method to a LARC, stating that since she did not have side effects it was not necessary.

#### Method Choice

Women reported a variety of experiences that led them to their choice of method. Some were first-time users, while others had tried a variety of methods before settling on their current one. This could be because of failure of a natural method or undesirable side effects with some hormonal methods.

IUD users appreciated that they were long-acting. Users who had chosen other methods appeared to have been dissuaded from choosing an IUD because of misinformation, and believed incorrectly that side effects of IUDs could include infections or cancer. One thought that an IUD would make her sterile or get lost inside her body. The method of insertion via the vagina and cervix discouraged other women from choosing an IUD over other methods.

Respondents reported that “Depo” was the most known method of contraception. It should be noted that several respondents spoke about “Depo” that was injected, “Depo for 3 years or 5 years that you put under the skin,” and that of 6 months that was injected in the neck, suggesting that the term “Depo” was loosely used to describe contraception in general and not just the injectable method of Depo-Provera. Nevertheless, several women stated they preferred the short duration of “Depo” because it allowed them to stop using it whenever they did not want to get pregnant again. For others, they chose an injectable method as a “test” before deciding to use a LARC.

Implant users appreciated the effectiveness and long-acting nature of the method, as well as that it could be removed at any time. A few noted its side effects. A few women also mentioned that they had not chosen CycleBeads because they were illiterate and would therefore find it difficult to use.

#### Secret Use

The vast majority of users did not want to share with other people that they were using family planning. They feared that other people would reveal their secret to the rest of the community. Some reported that only their family members, their neighbors, and friends knew. One woman explained, “It's a secret for me, my husband, and the nurse.” Others were open about their use and served as models in their community to sensitize others about family planning.

The vast majority of users did not want to share with other people that they were using family planning, but others were open about their use.

### Qualitative Data From Non-Users of Contraception

As explained in the Methods section, data in the following figures are salience index scores, which range from 0 to 1 and indicate the relative percentile rank of items within each topic area across the focus groups with male and female non-users.

#### Reasons for Adopting Family Planning

**FIGURE 4.**
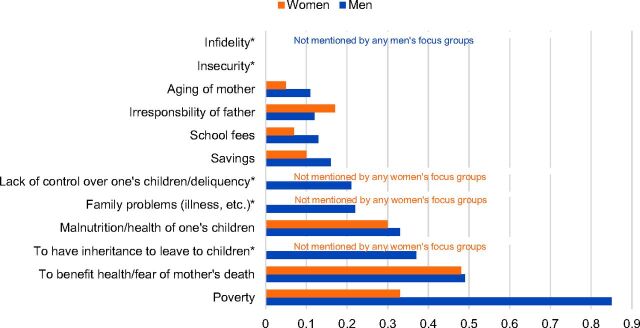
Salience Index Scores for Reasons People Might Accept Family Planning Cited by Male and Female Non-Users of Contraception Note: Salience index scores range from 0 to 1 and indicate the relative percentile rank of items within each topic area across the focus groups with male and female non-users. * Asterisked items indicate categories that were either not mentioned at all by the men's and/or women's focus groups or mentioned by at least one focus group but the salience scores approached zero. In this instance, inheritance, family problems, and lack of control over one's children were not mentioned by any of the women' focus groups, and infidelity was not mentioned by any of the men's focus groups.

Non-users cited several reasons why they thought people might choose to adopt family planning. Similar to users, poverty, health of the mother and risk of maternal mortality, as well as the health of existing children emerged among the most important reasons (Figure 4).

#### Reasons for Not Adopting Family Planning

**FIGURE 5.**
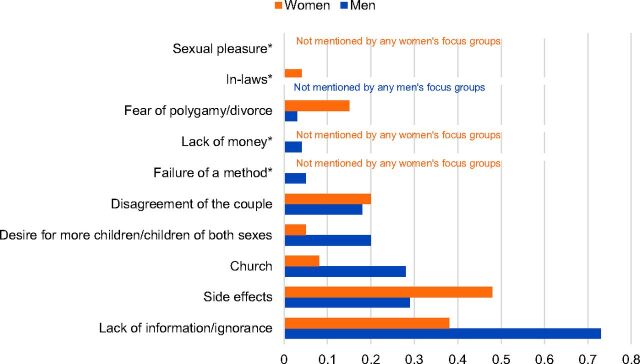
Salience Index Scores for Reasons People Might Not Adopt Family Planning Cited by Male and Female Non-Users of Contraception Note: Salience index scores range from 0 to 1 and indicate the relative percentile rank of items within each topic area across the focus groups with male and female non-users. * Asterisked items indicate categories that were either not mentioned at all by the men's and/or women's focus groups or mentioned by at least one focus group but the salience scores approached zero. In this instance, failure of a method, lack of money, and sexual pleasure were not mentioned by any of the women's focus groups, and in-laws was not mentioned by any of the men's focus groups.

For non-users, the lack of knowledge and perceived risk of side effects were cited as the primary obstacles to adopting modern methods of family planning (Figure 5). Myths about family planning, such as the risk of sterility or cancer and the need to have a cesarean section for subsequent pregnancies, were reported. Women also mentioned disagreements between spouses as an important barrier to family planning uptake. The participants discussed at length the paradox that children were extremely valued but at the same time acknowledged they were a financial burden.

#### Sources of Information

**FIGURE 6.**
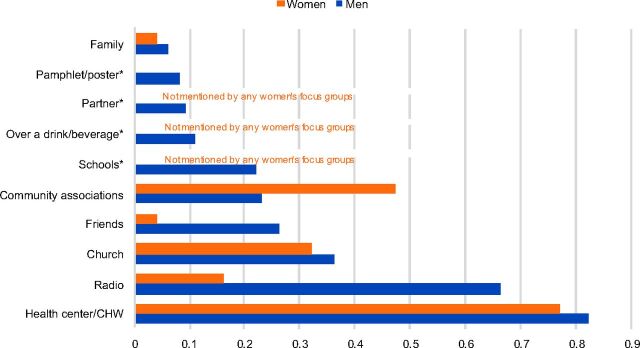
Salience Index Scores for Sources of Information About Family Planning According to Male and Female Non-Users of Contraception Abbreviation: CHW, community health worker. Note: Salience index scores range from 0 to 1 and indicate the relative percentile rank of items within each topic area across the focus groups with male and female non-users. * Asterisked items indicate categories that were either not mentioned at all by the men's and/or women's focus groups or mentioned by at least one focus group but the salience scores approached zero. In this instance, schools, over a drink/beverage, and partner were not mentioned by any of the women's focus groups.

According to non-users, health centers and collective activities such as antenatal care sessions were the primary sources of information about family planning services (Figure 6). Community sensitizations with megaphones and flipcharts were also important sources of information. The church played a prominent role in the diffusion of messages. Female non-users also mentioned local organizations or associations, religious communities, female leaders of associations, and discussions between women, including between friends while working in the fields or at the river. Although many women keep their family planning use secret, as noted earlier, the non-users expressed a desire for users to share their positive experiences with other women in the community. For men, the radio was an important source of information that could be used to share information on family planning.

Non-users expressed a desire for users to share their positive experiences with family planning with other women in the community.

#### Influential People in Decision Making

**FIGURE 7.**
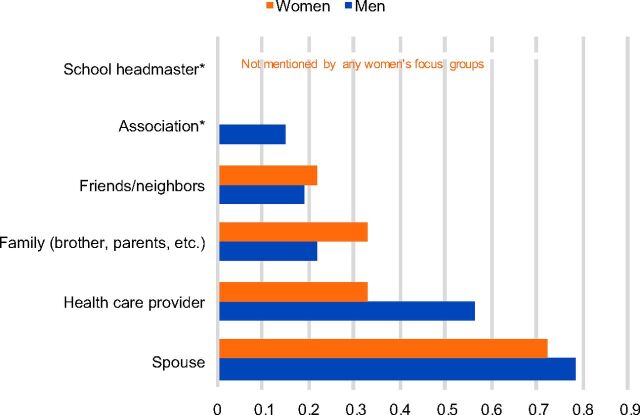
Salience Index Scores for Influential People in Making Decisions About Family Planning According to Male and Female Non-Users of Contraception Note: Salience index scores range from 0 to 1 and indicate the relative percentile rank of items within each topic area across the focus groups with male and female non-users. * Asterisked items indicate categories that were either not mentioned at all by the men's and/or women's focus groups or mentioned by at least one focus group but the salience scores approached zero. In this instance, school headmaster was not mentioned by any of the women's focus groups.

Respondents reported that decision making about family planning was done first between the man and the woman in the couple affected (Figure 7). In the majority of cases, it was the husband who made the final decision about using family planning, including the type of method if the decision was to use family planning. Cases where the woman made the decision alone were reportedly rare. If the woman chose a method that was not approved by her partner, respondents expressed that the woman should change the method or stop using it. Family members, including the children and friends, could also negatively or positively influence the decision of a woman.

#### Misconceptions, Rumors, and Misunderstandings

As noted earlier, many rumors and misconceptions about family planning surfaced during the interviews and FGDs. These included:

- A woman using an implant cannot work in the fields
- Contraception can cause permanent infertility, cancer, or epilepsy
- After using family planning, deliveries must be by cesarean section
- The second dose of an injectable contraceptive is long acting and a third dose is not needed
- Contraception is only for women living alone and condoms are only used by prostitutes
- Products such as aspirin, salty water, quinine, or strong liquor can be used as contraceptives
- Contraceptives reduce sexual pleasure
- An implant must be removed by the same provider who inserted it

Myths about IUDs in particular included that:

- IUDs cause infections that turn into cancer
- IUDs cause diabetes
- IUDs can get lost in the body, requiring surgical intervention
- IUDs are for permanent contraception
- An IUD is an injection in the vagina

Many rumors and misconceptions about family planning surfaced during the interviews and focus group discussions.

## PROGRAM MODIFICATIONS

As a result of our analysis of quantitative and qualitative data in 2013, we developed a set of recommendations and revised program activities (Table 2). The revised activities began in the 2 health zones in January 2014. Most of the supply-side activities targeting service quality issues identified in the formative research were based on findings that although health care providers were a primary source of information on family planning, there were gaps in provider knowledge and possibly in their competency. On the demand side, new activities targeted key influencers, information channels to address the gaps in knowledge, misconceptions, and rumors identified by the formative research.

**TABLE 2.** Key Program Strategies Before and After the Formative Assessment (New Activities in Bold)

	2011–2013	2014–2016	Formative Assessment Themes Addressed
Supply-side activities	Competency-based clinical training Provision of commodities and equipment Supportive supervision Support for data collection and use	Competency-based clinical training Provision of supplies and equipment Supportive supervision Support for data collection and use **Systematic clinical coaching and tracking individual provider competence** **Peer supervision by high-performing providers** **Values clarification activities with providers** **Introduction of LNG-IUS** **Introduction of Population Council's Balanced Counseling Strategy**	Quality of services Side effects
Demand-side activities	Large community meetings on the benefits and availability of family planning Home visits by community health workers that include discussion of family planning	**Large, multichannel SBCC campaigns on family planning** **Community mobilization by satisfied users** **Targeted outreach to male partners** **Actively dispelling rumors about certain methods** Home visits by community health workers that include discussion of family planning	Lack of knowledge; misconceptions, rumors, misunderstanding; sources of information Influential people in decision making

Abbreviations: SBCC, social and behavior change communication; LNG-IUS, levonorgestrel-releasing intrauterine system.

### Supply-Side Activities

#### Analysis and Discussion of Program Data at the Health Facility and Community Levels

The MoPH and IRC began analyzing family planning data and identifying community-specific program improvements alongside health care providers and community members in 2014. This enabled stronger ownership of program results at the health facility and community levels, which empowered community members and providers to increase uptake of family planning through several of the strategies described below.

#### Values Clarification Activities for Providers

The experiences of clients described during the key informant interviews revealed many misconceptions about IUDs among providers as well as a lack of high-quality family planning counseling. IRC's clinical supervisors integrated values clarification activities into routine supportive supervision visits and provider meetings in order to correct misconceptions and improve provider attitudes about IUDs. These activities included discussions meant to encourage provider reflection on the attitudes they held toward some family planning methods and the women and girls who use them.

#### Clinical Coaching by IRC and MoPH Supervisors

Prior to the formative research, clinical coaching of individual providers on key family planning competencies was ad hoc. In 2014, IRC improved the supportive supervision system so that providers received systematic, individual clinical coaching on each competency relevant to family planning using standardized checklists. The results of these coaching visits and provider competency scores were tracked over time and those providers with lower competency were prioritized for more intensive clinical coaching, and in some cases refresher training, as needed.

#### Peer Supervision with Higher-Performing Providers

During supportive supervision visits, IRC supervisors also identified clinical competency in IUD insertion and removal as a barrier to high-quality service delivery, which mirrored the formative findings on some providers' lack of knowledge about IUDs. We identified providers highly competent in IUD insertion and removal and empowered them to coach other providers at their facility and within their health zone to improve their skills. Supervisors reported that this system motivated both the peer supervisors as well as other providers to improve their skills in IUD insertion and removal.

Providers who were highly competent in IUD insertion and removal were empowered to coach other providers at their facility and within their health zone.

#### Introduction of Immediate Postpartum IUDs

To increase opportunities for adoption of family planning, IRC also began training and supporting providers to insert IUDs immediately postpartum in 2014. The introduction of this service further demonstrated the advantages of the IUD to both providers and clients. Anecdotally, training on postpartum IUD insertion indirectly improved provider perceptions of the method and the quality of counseling offered about the IUD.

#### Training on Population Council's Balanced Counseling Strategy Plus Approach

From 2011 to 2013, supportive supervision visits revealed that the content of family planning counseling primarily focused on the duration of efficacy of the methods and did not emphasize other important method characteristics, such as effectiveness, convenience of use, side effects, and hormone content. Providers often encouraged, directly or indirectly, clients to choose the method whose duration of efficacy matched their desired birth spacing duration, without adequately informing them of the ability to remove long-acting methods whenever the clients wished. This practice may have made clients less likely to choose the IUD because they wanted to space for fewer than 10 years. To improve the quality of family planning counseling, the organization trained all providers on Population Council's Balanced Counseling Strategy Plus approach.^18^ This strategy uses a standardized algorithm and job aids to guide family planning counseling sessions to ensure that clients receive all information that is relevant to their experience and needs, have an opportunity ask questions, and are referred to other reproductive health services, as needed.

Providers often encouraged clients to choose a method whose duration of efficacy matched their desired birth spacing duration.

#### Introduction of the Levonorgestrel-Releasing Intrauterine System

In 2015, IRC introduced the levonorgestrel-releasing intrauterine system (LNG-IUS), in addition to copper-bearing IUDs, to expand client choice of LARCs. IRC-trained providers offered counseling on both types of IUDs, in addition to the other methods, and emphasized the important attributes of each method.

**Figure:**
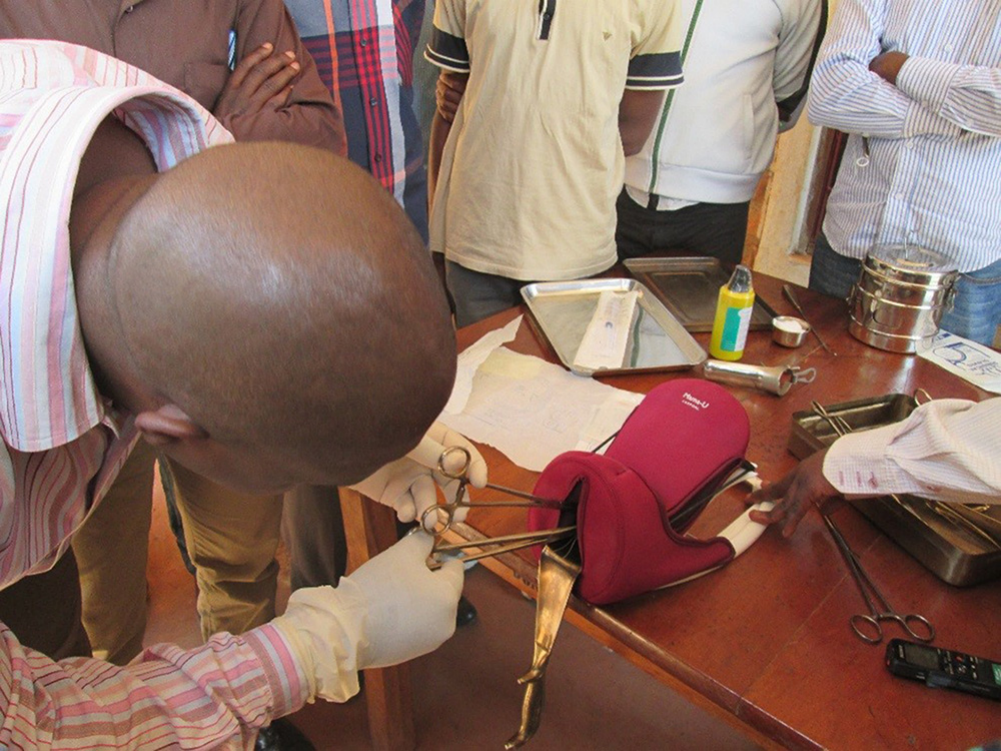
A Ministry of Public Health doctor demonstrates the postpartum IUD insertion technique to nurses and midwives in eastern DRC. © 2016/Mirindi Munyangura John/International Rescue Committee.

### Demand-Side Activities

#### Family Planning SBCC Campaigns

To increase demand for family planning services, we had already in 2013 begun conducting periodic, intensive SBCC campaigns to raise awareness about the availability and benefits of modern contraception. These 2-week SBCC campaigns were conducted twice each year in each health zone. IRC staff and volunteer community health workers conducted community events focused on family planning education, shared key family planning messages via motorized caravans and radio spots, and intensified home visits. These activities inundated the health zones with positive messages about modern contraception and available family planning services. The information from the formative research allowed further adaptation of the messaging and targeting during these SBCC campaigns, amplifying positive messages that resonated with beneficiaries such as the health of the mother and dispelling common myths such as the inability to reverse certain methods of contraception. The results of these SBCC campaigns are evident in the service statistics—the number of clients starting family planning methods increased an average of 2.5-fold during the months of the campaigns from 2014 to 2016, although the method mix did not change significantly during the months of the campaigns.

The number of clients starting family planning methods increased an average of 2.5-fold during the months of behavior change communication campaigns between 2014 and 2016.

#### Dispelling Rumors

IRC worked closely with the community health workers from the beginning of the project in June 2011, but we intensified our support for their activities beginning in 2014. Specifically, based on findings from the formative research IRC trained community health workers to actively identify rumors about specific methods, IUDs in particular. The community health workers then worked with IRC and the MoPH to create focused messages to dispel these rumors and integrated these messages into routine home visits conducted in supported health zones. Additionally, specific radio spots were developed and routinely aired to correct identified myths.

#### Community Mobilization by Satisfied Couples

Based on the desire of non-users in the formative research to hear the experiences of users firsthand, IRC identified couples who were happy with the method they chose and who were also willing to share their experience during community events. Recognizing that many clients were still unfamiliar with the IUD, those satisfied couples using the IUD were prioritized. These couples described their positive experience with the health facility and their method and encouraged others to seek family planning services during periodic community mobilization sessions and on the radio.

#### Outreach to Male Partners

Recognizing that male partners sometimes act as barriers to women's adoption of family planning and were identified as influential in decision making around family planning, IRC began targeting men with community mobilization activities in 2014. Specifically, IRC invited male partners to participate in community mobilization activities related to family planning at the maternity waiting homes (*binyolas*) and conducted family planning education activities at football games and local bars.

## RESULTS OF PROGRAM DESIGN CHANGES

### Quantitative Data on Users

**FIGURE 8.**
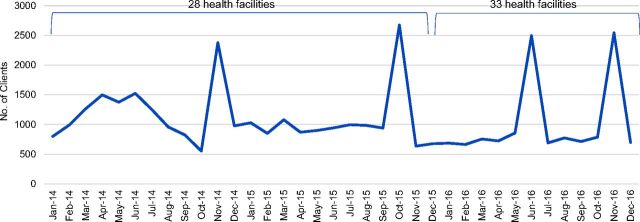
Number of Clients Adopting Family Planning Methods, Health Zones A and B in South Kivu, Democratic Republic of the Congo, Post-Program Design Changes, January 2014 to December 2016

A total of 39,399 clients started family planning methods at IRC-supported health facilities in South Kivu from January 2014 to December 2016, compared with just 8,985 from June 2011 to December 2013 (Figure 8). The mean number of clients who adopted family planning methods per month, per facility increased from 14 during the initial program period (June 2011 to December 2013) to 37 after the program was modified (January 2014 to December 2016)—representing a 64% increase. Peaks in uptake were observed at certain points—April 2014, May 2014, November 2014, October 2015, June 2016 and November 2016—as a result of extensive SBCC campaigns conducted during these months (Figure 8). The annual number of clients adopting family planning methods peaked in 2014 at 14,410, but declined to 12,590 in 2015 and 12,399 in 2016. Among 2,034 PAC clients treated from January 2014 to December 2016, 58% adopted family planning, compared with 29% of 1,100 PAC clients from June 2011 to December 2013.

The average number of clients who adopted family planning methods in each facility increased from 14 per month during the initial program period to 37 per month after the program was modified.

**FIGURE 9.**
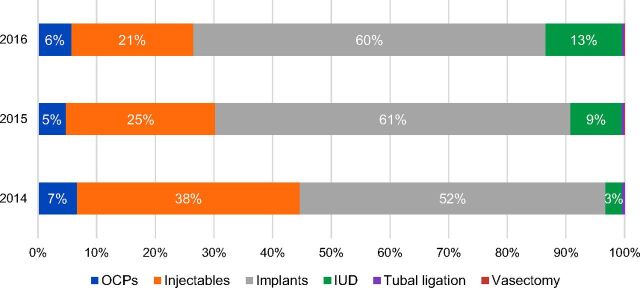
Trends in Contraceptive Method Mix Among Clients Starting Family Planning Methods, Health Zones A and B in South Kivu, Democratic Republic of the Congo, 2014–2016 Abbreviations: OCP, oral contraceptive pill; IUD, intrauterine device.

The percentage of clients adopting LARCs increased in both zones from 50% to 66% between the initial program period and the post-program design period. Implants continued to be the most popular method in both zones, reaching 61% of the method mix in 2015 and 60% in 2016 (Figure 9). While IUDs remained less popular than implants, 8% of clients in total adopted this method between 2014 and 2016. Notably, the percentage of clients adopting IUDs increased each year, from 3% in 2014 and reaching 13% of the method mix in 2016.

While IUDs remained less popular than implants, the percentage of clients adopting IUDs increased each year, from 3% in 2014 to 13% in 2016.

Among PAC clients, IUDs became the most popular method. Between 2014 and 2015, 45% of PAC clients who adopted family planning chose to use the IUD. Implants were the next most commonly adopted family planning method among PAC clients, followed by injectables, pills, and tubal ligation. This is in contrast to the June 2011 to January 2013 period, when implants were most popular method among PAC clients, followed by injectables, pills, IUDs, and tubal ligation.

## DISCUSSION

Through the Family Planning 2020 (FP2020) initiative, 38 countries to date have made ambitious commitments to increasing access to and use of family planning, with financial commitments expected to reach US$2.5 billion.^19^ The DRC has pledged to increase modern contraceptive prevalence from 6.5% to 19.0% by 2020, reaching an additional 2.1 million women with contraceptive services.^20^ As investment in providing access to contraception increases, it is important to understand how to deliver high-quality services most effectively and in a responsive way, particularly in conflict-affected areas where family planning is less likely to be prioritized or access to services may be disrupted. By using formative research and analysis of routine monitoring data, we were able to adapt our programming approach to address barriers to family planning uptake and client informed choice, such as provider bias, low clinical competency, lack of knowledge among users, and the influence of partners and other family members in family planning decision making. Our results challenge the frequent assumption that method mix reflects only client preference and demonstrates that program quality also plays an important role. Ensuring practical availability of a wide range of contraceptive methods through high-quality services not only increases choice but also increases overall uptake of family planning.^21^

Our results challenge the frequent assumption that method mix reflects only client preference and demonstrates that program quality also plays an important role.

The surges in family planning uptake seen after large SBCC campaigns—using communication channels suggested by study participants—are in line with the finding that lack of information may be an important reason why people are not using family planning. The widespread dissemination of positive messages about family planning during these campaigns may also have encouraged more discussion between couples and within the community, prompting women who had been considering using family planning to finally adopt a method. The large number of women adopting family planning during the SBCC campaigns also challenged the notion that the low number of clients adopting family planning each month in 2011 through 2013 reflected a lack of demand for services, a reason cited by some stakeholders at the time. Indeed, each SBCC campaign continued to demonstrate the high latent demand for family planning services in IRC-supported health zones. The experiences of family planning users suggested that women were often motivated to seek family planning to protect their own health and improve their economic situation after having multiple previous pregnancies.

A key component of improving informed choice for clients, along with increasing knowledge about family planning, was dispelling rumors and misconceptions that may have prevented users from adopting family planning and providers from offering the full range of methods. Furthermore, the increased frequency and quality of clinical coaching on family planning skills during supportive supervision beginning in 2014 played an important role in increasing provider confidence to offer long-acting methods, particularly the IUD. This experience suggests that formal, competency-based training alone is not sufficient to ensure provider competency, particularly in contexts where beneficiaries are unfamiliar with certain methods, such as the IUD in South Kivu. This unfamiliarity with certain methods results in low initial client load so that providers do not receive sufficient practice with real clients during or after trainings, perpetuating a lack of confidence and clinical quality that inhibits client informed choice.

We can see that uptake of family planning increased after the new program strategies were introduced—fewer than 9,000 clients adopted family planning methods during the first 2.5 years of the program while nearly 15,000 clients started family planning in 2014 alone. While this increase was in part due to an increase in the number of facilities supported by the project, the average number of adopters per facility, per month more than doubled, suggesting that the new program strategies also played an important role. Importantly, the percentage of PAC clients adopting family planning also doubled during the 2014–2016 period as compared with the 2011–2013 period. It is important to note that the definition used for the number of PAC clients adopting family planning did vary, which limits the comparability of these data over time. However, the annual number of clients starting family planning declined somewhat in both 2015 and 2016. This decline suggests that additional strategies are needed to sustain annual increases in clients adopting family planning methods.

The percentage of clients adopting long-acting methods also increased after implementation of new program strategies. Notably, the percentage of clients who adopted IUDs increased from just 2% in 2011 through 2013 to 13% in 2016. This increased uptake of IUDs was particularly notable among PAC clients who adopted family planning, among whom the percentage who adopted IUDs increased from 9% during the 2011–2013 period to 45% during the 2014–2016 period. Efforts to improve the quality of services may have both improved client informed choice and increased uptake by giving women more options. It is likely that the introduction of the LNG-IUS and the option of postpartum IUD insertion also contributed to the increase in uptake of IUDs. Anecdotally, clients appreciated how the LNG-IUS reduced bleeding and that it is shorter acting that the copper-bearing IUD. Though all clients are informed that they can remove their method any time, many clients continue to associate length of action with their desired duration of birth spacing. These results suggest that the low percentage of clients adopting IUDs early in the project was due, at least in part, to gaps in program quality rather than only client preference.

The increased uptake of IUDs was particularly notable among postabortion care clients.

Despite increases in adoption of IUDs, the skew toward implants increased, reaching 60% of the method mix in 2015 and 2016. Given the enhanced program interventions implemented to improve client informed choice, these results suggest that the trend toward implants may not have been due to issues with program quality. One explanation for these results is that method skew may be self-reinforcing once a particular method becomes dominant within a population. While not rated most salient, both men and women reported that friends and family were important sources of information as well as influential in decision making about family planning. Our results and previous research^22^^–^^26^ demonstrate that women and couples are indeed influenced by the opinions of friends and family when deciding whether to use contraception and which method to choose. In places where most women are adopting one particular method, these women may influence the contraceptive choices of their peers and family members, further reinforcing adoption of the dominant method. Supply-side barriers may continue to play a role, as well. In particular, frequent provider turnover is a constant challenge for the program as new providers must receive intensive support to ensure their competency and unbiased service provision.

The number of clients adopting family planning is an important indicator of service access.^27^ However, research demonstrates that, on average, nearly 40% of women discontinue use of their method within 12 months, many for reasons other than the desire to become pregnant. This “leaking bucket” phenomenon increases unmet need for family planning.^28^^–^^30^ Notably, contraceptive discontinuation rates tend to be much lower for women who adopt the IUD.^30^ These results from the DRC do not include data on contraceptive continuation and the contraceptive continuation rate for the program is unknown. This gap, as well as the fact that these data are not representative of the population, limit our ability to speculate about whether the program reduced unmet need for contraception.

### Limitations

The data presented are from routine program monitoring activities and were not intended as research. Therefore there are no control groups or population-based survey data that can show changes in contraceptive prevalence specifically in the organization's program area. Data on prevalence and method mix in other parts of DRC since 2013 are not readily available for purposes of comparison. In addition, the qualitative data come from a limited number of facilities in only 1 province in the DRC. For the non-users, the inclusion criteria of having at least 5 children meant that younger non-users were often not included, although our monitoring data show that uptake was in general lower among older women, which is why we chose these criteria. In addition, the selection of non-users may not have been representative even within that age range because they were purposively selected from community groups. This limits the conclusions that can be drawn about the impact of the project activities, although we believe the description of the processes can be transferable to similar programs in other areas. No other actors were supporting family planning or general primary health care programming in the 2 health zones that might have meaningfully contributed to the increases observed. We have observed similar improvements in uptake and client informed choice in other project-supported health zones across the DRC after implementation of the recommendations of the formative research.

## CONCLUSION

Although there has been increased attention to improving access to voluntary contraception globally, it is often overlooked in conflict-affected settings. Our experience in eastern DRC demonstrates that women and their partners affected by conflict want family planning, and that it is feasible to deliver the full range of modern contraceptive methods when programs are adapted and sensitive to the local context. Program monitoring data combined with formative research can identify areas for program improvement and strategies to address barriers to uptake in general and ensure client informed choice. Barriers are often found on both the demand and supply side, so programs should include components to address both.

## Notes

**Peer Reviewed**

**Funding:** Anonymous private foundation.
